# Prognostic prediction of systemic immune-inflammation index for patients with gynecological and breast cancers: a meta-analysis

**DOI:** 10.1186/s12957-020-01974-w

**Published:** 2020-08-07

**Authors:** Yongfang Ji, Haiyan Wang

**Affiliations:** 1 Department of Gynecology, Mengyin County People’s Hospital, No. 368 Dongmeng Road, Linyi City, 276299 Shandong Province China; 2 Department of Obstetrics, Mengyin County People’s Hospital, Linyi City, 276299 Shandong Province China

**Keywords:** Gynecological cancer, Breast cancer, Systemic immune-inflammation index, Prognosis

## Abstract

**Background:**

Systemic immune-inflammation index (SII) has been suggested to be effective to reflect the inflammatory status and thus may be an underlying biomarker for prognosis prediction. This hypothesis has been demonstrated in meta-analyses on several cancer types. However, there was no study to confirm the prognostic roles of SII for gynecological and breast cancers, which was the goal of our study.

**Methods:**

PubMed, EMBASE, and Cochrane Library databases were searched to collect the articles exploring the associations of SII with prognostic outcomes [overall survival (OS), disease-free survival (DFS), progression-free survival (PFS), lymph node metastasis (LNM), and lymphovascular invasion (LVI)] in gynecological and breast cancers. The prognostic value of SII was estimated by hazard ratio (HR) or relative risk (RR) with 95% confidence interval (CI).

**Results:**

Nine articles involving 2724 patients in 11 datasets were included. Meta-analysis showed that a high SII index was significantly associated with poor OS (HR = 2.12, 95% CI, 1.61–2.79, *P* < 0.001), DFS/PFS (HR = 2.28, 95% CI 1.52–3.41, *P* < 0.001) and an increased risk for LNM (RR = 1.34, 95% CI 1.20–1.50, *P* < 0.001) in patients with gynecological and breast cancers. Subgroup analysis confirmed the prognostic role of SII for OS was applicable to all cancer types, but the association with DFS/PFS and LNM was only significant for ovarian cancer and breast cancer, especially triple-negative breast cancer. No significant association was detected between SII and LVI.

**Conclusion:**

High SII may be a promising indicator for the prediction of poor prognosis in patients with gynecological and breast cancers, especially ovarian cancer and triple-negative breast cancer.

## Background

Gynecological and breast cancers are the two leading causes of death among women [1]. According to the epidemiological investigation in the USA in 2019, breast cancer was responsible for 41,760 deaths, followed by ovarian cancer (13,980), uterine corpus endometrial cancer (12,160), cervical cancer (4250), and vulvar cancer (1280) [2]. Recurrence and metastasis are the main contributors for the treatment failure and poor outcomes of these gynecological and breast cancer patients. Therefore, it may be a pivotal issue to identify the patients at a high risk of unfavorable prognosis in order to early schedule individualized preventive and therapeutic strategies.

In recent years, increasing evidence has shown that activation of inflammation is a crucial mechanism for the recurrence and metastasis of gynecological [3, 4] and breast [5, 6] cancers. Thus, inflammatory-related peripheral cells measured in routine blood test (such as neutrophils, lymphocytes, and platelets) and their derived index [including neutrophil-lymphocyte ratio (NLR), platelet-lymphocyte ratio (PLR), and systemic immune-inflammation index (SII, platelet count × neutrophil count/lymphocyte count)] may be potential prognostic biomarkers for gynecological and breast cancers. This hypothesis had been demonstrated by previous studies, especially for NLR and PLR [7–10]. Their prognostic values had been confirmed by an integrated meta-analysis of all updated evidence, that is, elevated NLR or PLR was associated with poor overall survival (OS) and disease-free survival (DFS) of patients with gynecological [7, 8] or breast [9, 10] cancer. For SII, only individual literatures were reported to reveal its prognostic ability for gynecological and breast cancers. For example, a retrospective study in ovarian cancer patients showed that SII was an independent prognostic indicator for OS and progression-free survival (PFS) not only in the training cohort [OS: hazard ratio (HR) = 6.36, 95% confidence interval (CI) = 2.64–15.33; *P* < 0.001; PFS: HR = 7.61, 95%CI = 3.34–17.35; *P* < 0.001], but also in the discovery cohort (OS: HR = 1.96, 95%CI = 1.09–3.63; *P* = 0.024; PFS: HR = 2.71, 95%CI = 1.48–4.93; *P* = 0.001) [11]. Multivariate analysis proved that increased SII correlated with poor OS (Liu et al.: HR = 2.60, 95% CI = 1.74–3.88; *P* < 0.001 [12]; Wang et al.: HR = 2.96, 95% CI = 2.18–3.98; *P* < 0.00 1[13]) and DFS (Liu et al.: HR = 1.46, 95% CI = 1.01–2.12; *P* = 0.045 [12]; Wang et al.: HR = 2.85, 95% CI = 1.62–3.81; *P* = 0.005 [13]) in patients with triple-negative breast cancer. Using primary (HR = 2.53, 95% CI = 1.32–4.83; *P* = 0.005) and validation (HR = 3.99, 95% CI = 1.388–11.47; *P* = 0.010) cohorts, the study of Huang et al. supported that SII was an independent risk factor for prediction of OS in cervical cancer patients [14]. Furthermore, receiver operating characteristics curve analysis suggested that the prognostic accuracy of SII for 5-year OS in patients with cervical cancer was even higher than NLR [area under the curves (AUC): 0.64 vs 0.59, primary; 0.64 vs 0.59, validation] or PLR (AUC: 0.64 vs 0.60, primary; 0.64 vs 0.60, validation) [14], indicating SII may represent a promising biomarker for predicting survival of gynecological cancer patients clinically. However, the associations between SII and clinical outcomes of gynecological and breast cancer patients were found to be nonsignificant in some other studies [15, 16]. Therefore, it is necessary to re-assess the prognostic value of SII in patients with gynecological and breast cancer patients by performing a meta-analysis like NLR and PLR, which was not reported previously and was the goal of this study.

## Materials and methods

This meta-analysis was performed based on the Preferred Reporting Items for Systematic Review and Meta-Analysis (PRISMA) statement. Because the present study was a meta-analysis of articles published previously, ethical approval and patient consent were not required.

### Search strategy

Two authors independently searched eligible articles in PubMed, EMBASE, and Cochrane Library from the date of establishment to January 1, 2020. The search strategy included (“gynecological” OR “breast” OR “cervical” OR “ovarian” OR “endometrial”) AND (“cancer” or “carcinoma” or “tumor”) AND (“systemic immune-inflammation index” or “SII”). Additionally, references of included publications and reviews were manually reviewed for potential trials.

### Inclusion and exclusion criteria

The study selection was completed by two independent investigators. Publications were eligible if they met the following inclusion criteria: (1) the enrolled patients suffered from gynecological and breast cancers which were pathologically diagnosed; (2) patients did not have an active infection, inflammatory, or comorbid diseases or undergo anti-inflammatory medication before blood examination; (3) neutrophil, platelet, and lymphocyte counts were measured prior to any treatments and SII was calculated; (4) the associations between SII and prognostic outcomes of patients were assessed; (5) the HRs with their 95%CIs were reported or could be calculated from raw data; (6) the cut-off value of SII was provided; and (7) articles were published in English. The exclusion criteria were as follows: (1) studies were duplicated or data were overlapped; (2) letters, case reports, editorials, or reviews; (3) non-human studies; and (4) insufficient data for estimating HRs and 95%CIs for prognosis outcomes.

### Data extraction and quality assessment

Two authors extracted the following data independently: the name of the first author, year of publication, country, sample size, cancer type, study design, treatment, follow-up period, SII cut-off, source of HRs, outcomes, and HR with 95%CI for each outcome. HR based on multivariate analysis was preferentially extracted if available. The Newcastle-Ottawa Scale (NOS) criteria [17] was used by two independent researchers to evaluate the quality of enrolled studies, with scores ≥ 6 suggested to be of high quality.

### Statistical analysis

The HR and 95%CI of each study were calculated using STATA 13.0 (STATA Corporation, College Station, TX, USA) to assess the associations of SII with OS, PFS, and DFS. The relative risk (RR) and 95%CI were calculated for lymph node metastasis (LNM) and lymphovascular invasion (LVI). A pooled HR or RR > 1 indicated a poor prognosis for patients with high SII. Statistical difference was determined by using a *z* test (*P* < 0.05) with 95%CI (range not including the value of 1). Cochrane’s *Q* and *I*^2^ statistic tests were used to measure the heterogeneity of included studies. *P* < 0.10 and *I*^2^ > 50% indicated the presence of heterogeneity among studies, so that the pooled HR was calculated by a random-effects model; otherwise, a fixed-effects model was applied. Subgroup analyses were also performed by country, sample size, cut-off, cancer type, follow-up length, and source of HR. The values for dividing the subgroup of sample size, cut-off, and follow-up were selected according to the integer value of the median. Publication bias was estimated by Egger’s linear regression test (*P* < 0.05 indicated a significant publication bias) [18]. Publication bias was adjusted using the trim-and-fill procedure [19]. The robustness of the results was assessed by sensitivity analysis in which each study was removed in turn.

## Results

### Study characteristics

**Fig. 1:**
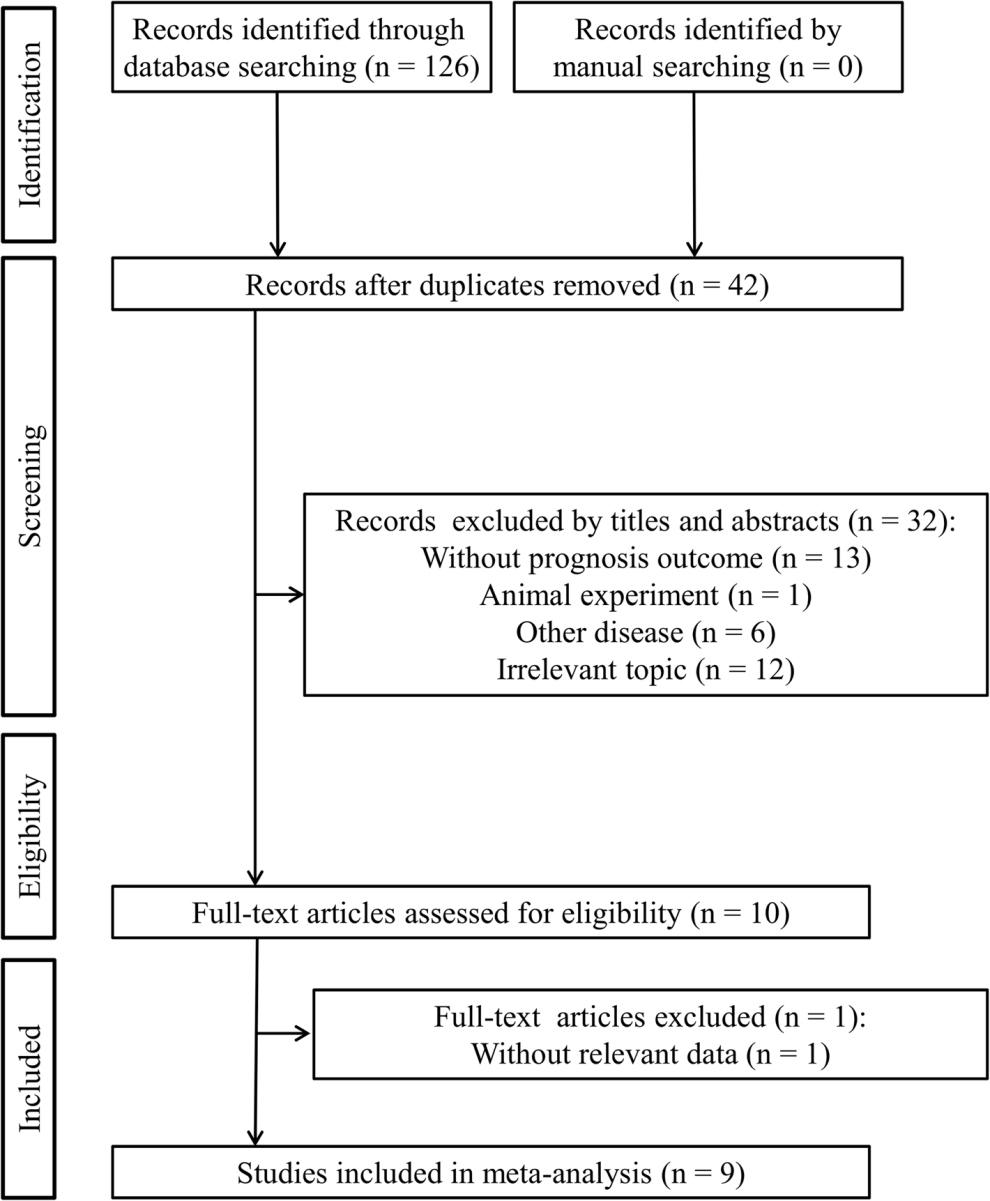
Flow diagram of included studies

As shown in Fig. 1, a total of 126 records were initially yielded through an electronic search on online databases. After removing duplicates (*n* = 84) and screening titles and abstracts (*n* = 32), 10 studies were assessed by full text for eligibility. One study was excluded due to lack of relevant data. No additional records were identified through manual searching. Finally, 9 published articles involving 2724 patients were included in this meta-analysis [11–16, 20–22]. Among these 9 studies, two [11, 14] contained the training and validation cohorts from different hospitals, and thus, 11 datasets were totally used for statistical analysis (Table 1). Two studies [14, 20] evaluated the relationship between SII index and prognosis in cervical cancer patients, five [12, 13, 16, 21, 22] focused on breast cancer, and two [11, 15] investigated ovarian cancer. In the study of De Giorgi et al., triple-negative, HER2+, and HER2–ER+ subtypes of breast cancer were also independently analyzed, in addition to the overall results [16]; while all the other studies [12, 13, 21, 22] on breast cancer only focused on specific breast cancer subtypes. The endpoint was OS in eight studies and PFS/DFS in six studies. Furthermore, the association of the SII index with LNM and LVI was also reported in five [11, 13, 14, 21, 22] and two [14, 22] studies, respectively. All these studies were retrospectively performed in China (*n* = 6), Italy (n = 1), USA (*n* = 1), or Spain (*n* = 1). Most of the studies (7/9, 77.8%) extracted the HR and 95%CI from the multivariate analysis and only 2 from the univariate analysis [16, 20]. The SII cut-off values ranged from 475 to 1000. The other characteristics of all cohort studies could be seen in Table 1. The NOS was 9 for six articles, 8 for two studies, and 7 for one study, indicating the included literature was overall of high-quality (Table 1).

**Table 1:** Characteristics of included studies

Study	Year	Country	No.	Cancer type	Design	Follow-up	Cut-off	Outcome	Treatment	HR source	NOS
Huang [14]	2019	China	458 (328 [training] + 130 [validation])^a^	Cervical cancer (FIGO stage I, II)	R, multi-center	47 m	475	OS, LNM, LVI	Surgery	M	9
Nie [11]	2019	China	533 (250 [training] + 283 [validation])^a^	Ovarian cancer (FIGO stage I-IV)	R, multi-center	46 m	612	OS, PFS, LNM	Surgery	M	9
Farolfi [15]	2018	Italy	375	Ovarian cancer (FIGO stage III-IV)	R, multi-center	43 m	730	OS, PFS	Chemotherapy	M	7
De Giorgi [16]	2019	USA	516	Breast cancer (triple-negative, HER2+, HER2– ER+)	R, single-center	–	836	OS	Systemic treatment	Overall (U), subtype (M)	8
Liu [12]	2019	China	160	Triple-negative breast cancer	R, single-center	61.7 m	557	OS, DFS	Surgery, chemotherapy, radiotherapy	M	8
Sun [21]	2019	China	155	Hormone receptor-HER2 + breast cancer	R, single-center	57.6 m	578	OS, DFS, LNM	Surgery, chemotherapy, radiotherapy	M	9
Li [22]	2019	China	161	Luminal breast cancer	R, single-center	28.4 m	518	DFS, LNM, LVI	Surgery, chemotherapy, radiotherapy, endocrine therapy	M	9
Wang [13]	2019	China	215	Triple-negative breast cancer	R, single-center	49.2 m	624	OS, DFS, LNM	Surgery, chemotherapy, radiotherapy	M	9
Holub [20]	2019	Spain	151	Cervical cancer (FIGO stages I–IV)	R, single-center	43.8 m	1000	OS	Surgery, chemotherapy, radiotherapy	U	9

*FIGO* International Federation of Obstetrics and Gynecology, *HER2* epidermal growth factor receptor type 2 *ER* estrogen receptor, *R* retrospective, *m* month, *OS* overall survival, *PFS* progression-free survival, *DFS* disease-free survival, *DMFS* distant metastasis-free survival, *LNM* lymph node metastasis, *LVI* lymphovascular invasion, *HR* hazard ratio, *M* multivariate, *U* univariate

^a^Including the training and validation cohorts

### Meta-analysis for OS

**Fig. 2:**
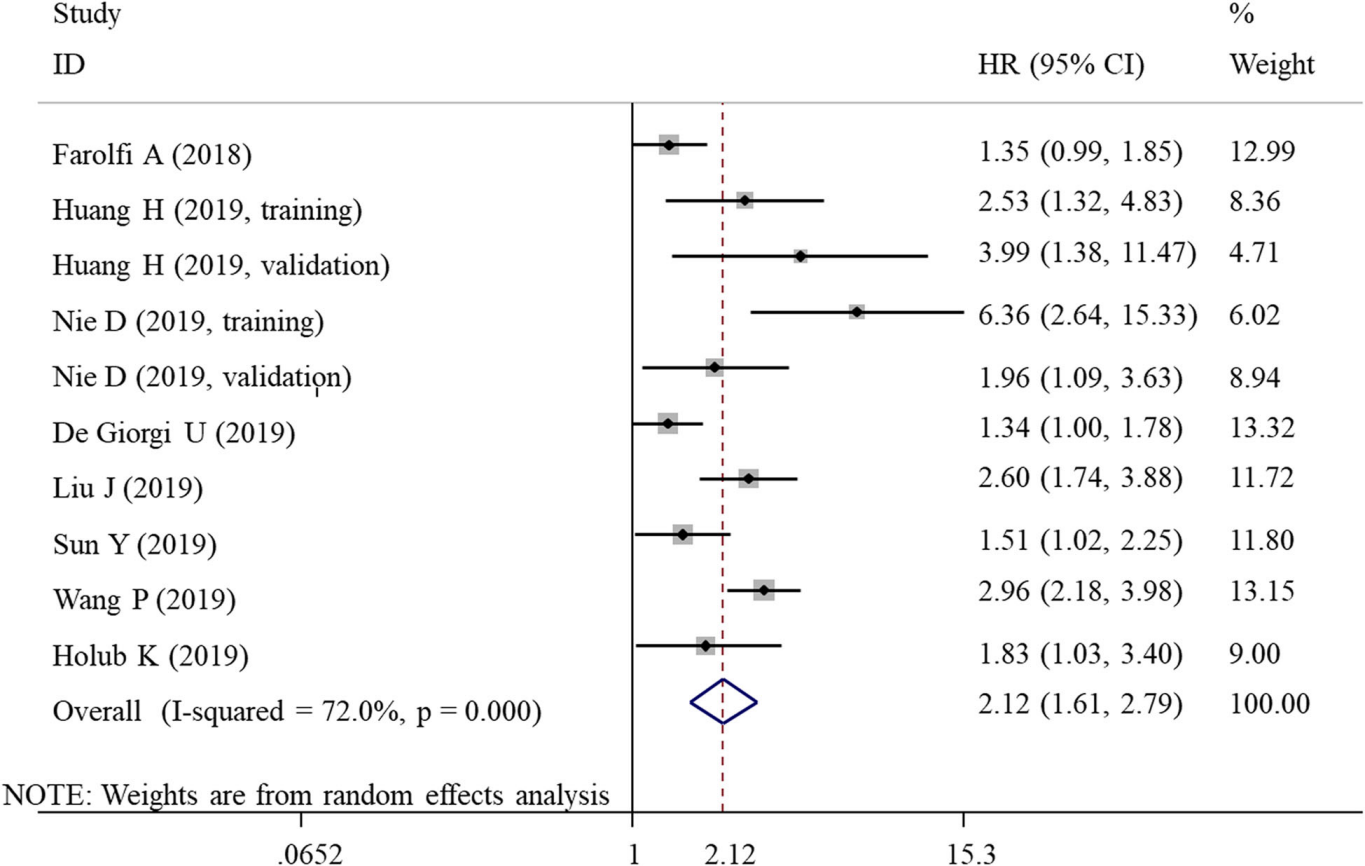
Forest plots showing the association between SII and overall survival. SII, systemic immune-inflammation index; HR, hazard ratio; CI, confidence interval

As there was obvious heterogeneity among the eight studies with ten datasets, the random-effects model was used (*I*^2^ = 72.0%, *P* < 0.001). The pooled results indicated that a high SII index was significantly associated with shorter OS in patients with gynecological and breast cancers (HR = 2.12, 95% CI = 1.61–2.79; *P* < 0.001) (Fig. 2). In order to explore the potential source of heterogeneity, subgroup analysis was conducted by country, sample size, cut-off, cancer type, follow-up length, and source of HR. The results demonstrated that these subgroup factors did not change the prognostic roles of SII index for OS (Table 4), with HR > 1 and *P* < 0.05 for all subgroups (Table 2).

**Table 2:** Meta-analysis for OS

Comparison		Studies	HR (95%CI)	*P*_A_ value	*I*^2^	*P*_H_-value	Model
Overall		10	2.12 (1.61,2.79)	< 0.001	72.0	< 0.001	R
Subgroup							
Country	Asian	7	2.56 (1.90, 3.44)	< 0.001	54.5	0.040	R
	Non-Asian	3	1.39 (1.14, 1.70)	0.001	0.0	0.634	F
Sample size	< 200	4	2.08 (1.46, 2.96)	< 0.001	43.3	0.152	F
	> 200	6	2.16 (1.44, 3.22)	< 0.001	81.2	0.000	R
Cut-off	< 600	4	2.24 (1.54, 3.23)	< 0.001	45.2	0.140	F
	> 600	6	2.05 (1.39, 3.04)	< 0.001	80.5	< 0.001	R
HR source	M	8	2.34 (1.70,3.21)	0.000	77.1	0.001	R
	U	2	1.42 (1.10,1.84)	0.008	0.0	0.357	F
Cancer type	Cervical cancer	3	2.34 (1.55, 3.50)	< 0.001	0.0	0.431	F
	Ovarian cancer	3	2.33 (1.08, 5.04)	0.032	81.7	0.004	R
	Breast cancer	4	1.98 (1.31, 2.99)	0.001	82.8	0.001	R
	TNBC	3	2.16 (1.31,3.56)	0.002	78.7	0.009	R
	Other BC type	3	1.79 (1.29, 2.49)	0.005	0.0	0.863	F
Follow-up	< 48 m	7	2.42 (1.66, 3.52)	< 0.001	70.9	0.002	R
	> 48 m	2	1.98 (1.16, 3.37)	0.012	72.0	0.059	R
	Unclear	1	1.34 (1.00, 1.79)	0.047	–	–	R

*TNBC* triple-negative breast cancer, *OS* overall survival, *m* month, *HR* hazard ratio, *CI* confidence interval, *M* multivariate, *U* univariate, *R* random-effects, *F* fixed-effects;*P*_*A*_*P* value for association; *P*_H_, *P* value for heterogeneity

### Meta-analysis for DFS/PFS

**Fig. 3:**
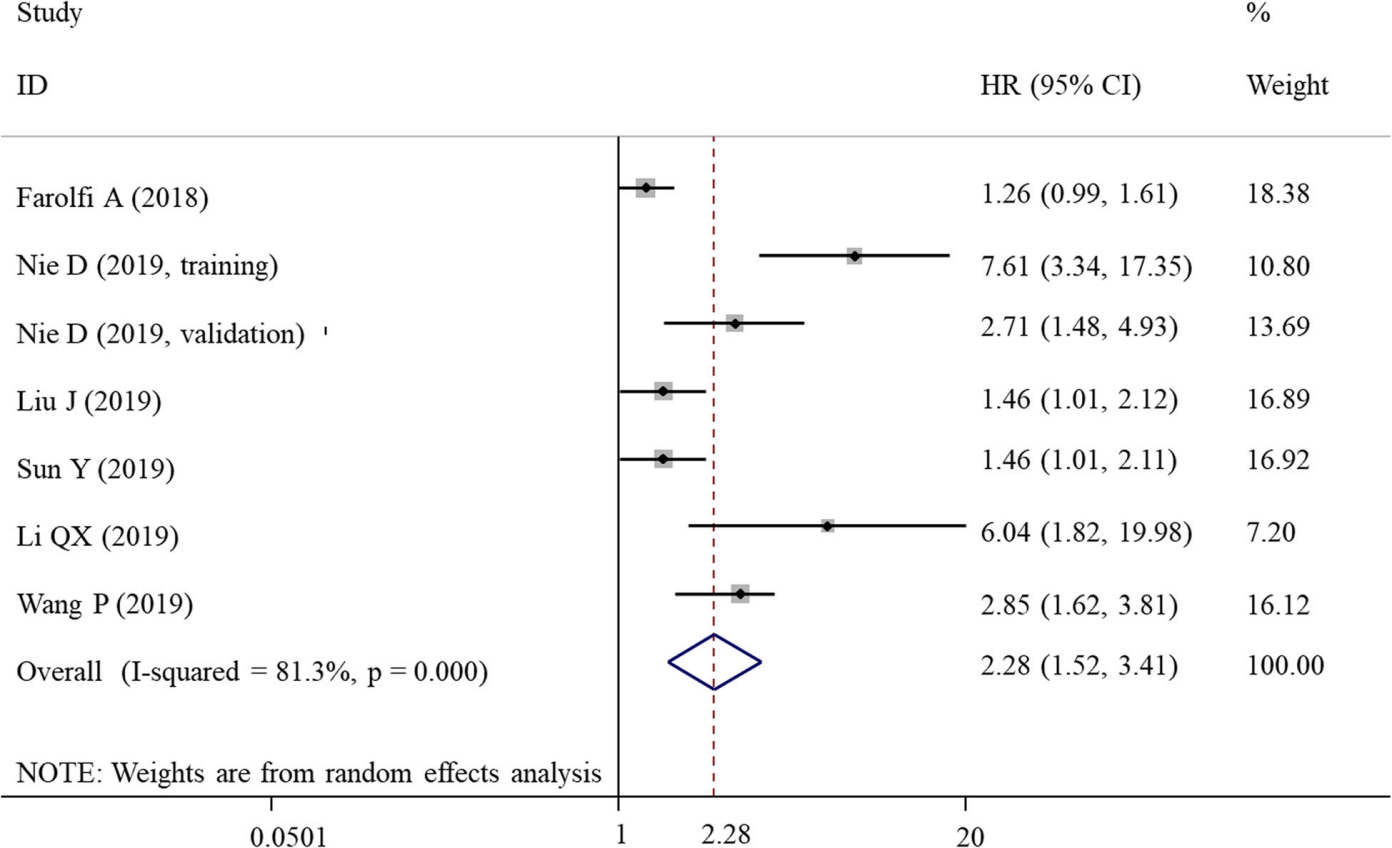
Forest plots showing the association between SII and disease-free survival/progression-free survival. SII, systemic immune-inflammation index; HR, hazard ratio; CI, confidence interval

The PFS was integrated with DFS for the meta-analysis as these outcomes are similar. The random-effects model was used to analyze the prognostic value of SII index for DFS/PFS because significant heterogeneity was present (*I*^2^ = 81.3%, *P* < 0.001) (Table 3). The meta-analysis revealed that a high SII index was a negative predictor of DFS/PFS for patients with gynecological and breast cancers (HR = 2.28, 95% CI = 1.52–3.41; *P* < 0.001) (Fig. 3). This prognostic significance of SII index was also confirmed in subgroup analyses according to country (Asian, *P* < 0.001), sample size (< 200, *P* = 0.022; > 200, *P* = 0.004), cut-off (< 600, *P* = 0.022; > 600, *P* = 0.004), cancer type (ovarian cancer, *P* = 0.042; overall breast cancer, *P* = 0.002; triple-negative breast cancer, *P* = 0.035), and follow-up length (< 48 months, *P* = 0.010; > 48 months, *P* = 0.005) (Table 3).

**Table 3:** Meta-analysis for DFS/PFS

Comparison		Studies	HR (95%CI)	*P*_A_ value	*I*^2^	*P*_H_ value	Model
Overall		7	2.28 (1.52, 3.41)	< 0.001	81.3	< 0.001	R
Subgroup							
Country	Asian	6	2.63 (1.65, 4.17)	< 0.001	77.7	0.000	R
	Non-Asian	1	1.26 (0.99, 1.61)	0.062	–	–	R
Sample size	< 200	3	1.74 (1.08, 2.80)	0.022	61.2	0.076	R
	> 200	4	2.74 (1.37, 5.46)	0.004	88.6	0.000	R
Cutoff	< 600	3	1.74 (1.08, 2.80)	0.022	61.2	0.076	R
	> 600	4	2.74 (1.37, 5.46)	0.004	88.6	0.000	R
Cancer type							
	Cervical cancer	–	–	–	–	–	–
	Ovarian cancer	3	2.78 (1.04, 7.45)	0.042	90.2	0.000	R
	Breast cancer	4	2.05 (1.30, 3.24)	0.002	72.3	0.013	R
	TNBC	2	2.02 (1.05, 3.89)	0.035	81.4	0.021	R
	Other BC type	2	2.64 (0.67, 10.39)	0.166	79.7	0.026	R
Follow-up							
	< 48 m	4	2.42 (1.66, 3.52)	0.010	88.0	0.000	R
	> 48 m	3	1.98 (1.16, 3.37)	0.005	70.8	0.033	R

*TNBC* triple-negative breast cancer, *PFS* progression-free survival, *DFS* disease-free survival, *m* month, *HR* hazard ratio, *CI* confidence interval, *R* random-effects, P_A_*P* value for association, P_H_*P* value for heterogeneity

### Meta-analysis for LNM

**Fig. 4:**
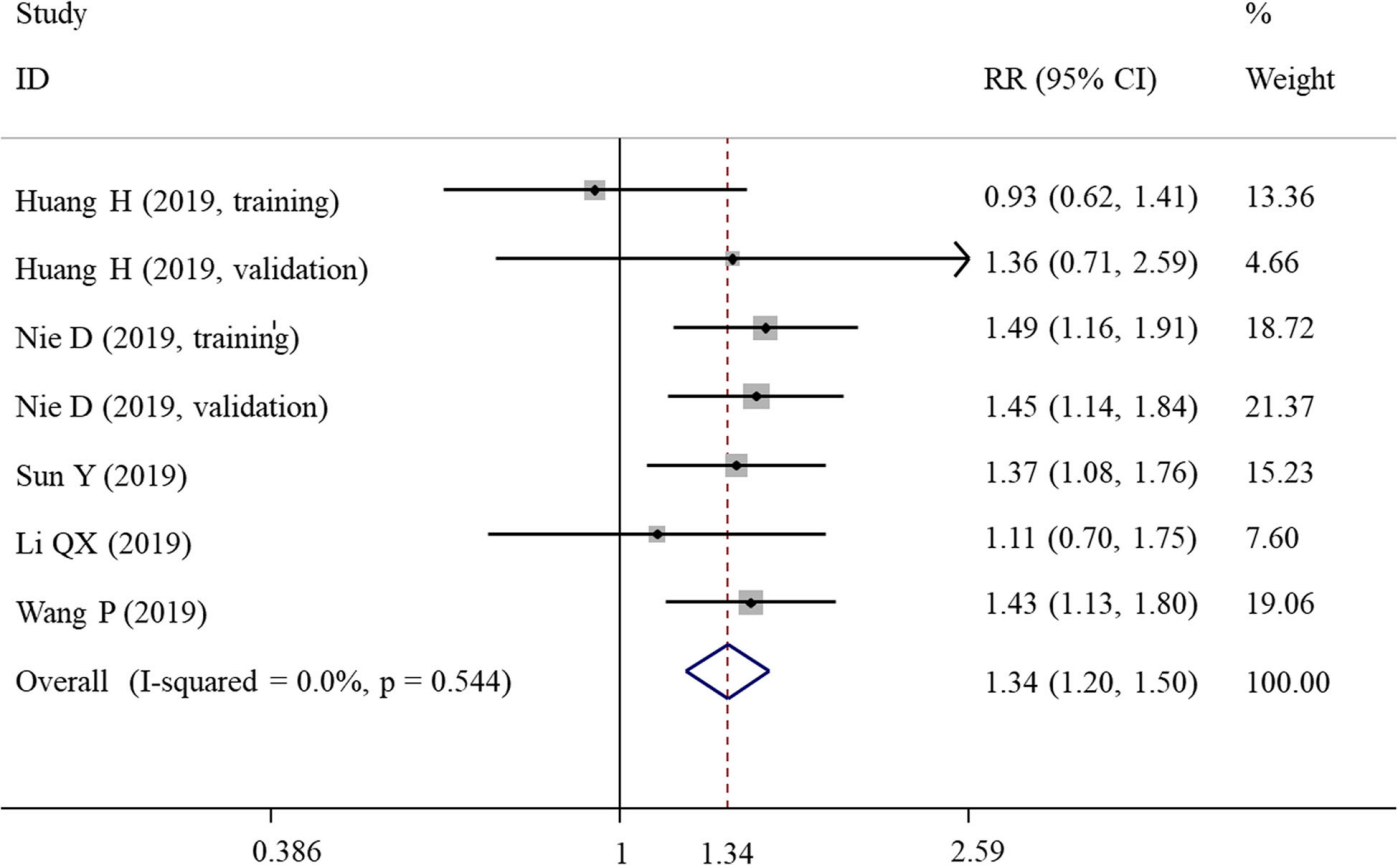
Forest plots showing the association between SII and lymph node metastasis. SII, systemic immune-inflammation index; HR, hazard ratio; CI, confidence interval

There was no heterogeneity observed among studies (*I*^2^ = 0%, *P* = 0.544); therefore, a fixed-effects model was used (Table 4). As shown in Fig. 4, the patients with a high SII index were at a significantly increased risk of LNM compared with those with a low SII index (RR = 1.34, 95% CI = 1.20–1.50; *P* < 0.001) (Fig. 4). Subgroup meta-analysis showed that the prognostic role of SII was only significant for patients with ovarian cancer and breast cancer (regardless of subtype), but not for cervical cancer (*P* = 0.807). Furthermore, in the subgroup with cut-off less than 600, the risk differences of LNM were not statistically significant between high and low SII index (*P* = 0.094) (Table 4).

**Table 4:** Meta-analysis for lymph node metastasis

Comparison		Studies	RR (95%CI)	*P*_A_ value	*I*^2^	*P*_H_ value	Model
Overall		7	1.34(1.20, 1.50)	< 0.001	0.0	0.544	F
Subgroup							
Country	Asian	7	1.34(1.20, 1.50)	< 0.001	0.0	0.544	F
	Non-Asian	0	–	–	–	–	–
Sample size	< 200	3	1.30(1.05, 1.61)	0.017	0.0	0.707	F
	> 200	4	1.36(1.19, 1.55)	< 0.001	27.3	0.248	F
Cut-off	< 600	4	1.18(0.97, 1.43)	0.094	0.0	0.393	F
	> 600	3	1.46(1.27, 1.67)	< 0.001	0.0	0.972	F
Cancer type	Cervical cancer	2	1.04(0.74, 1.48)	0.807	0.0	0.338	F
	Ovarian cancer	2	1.47(1.23, 1.75)	< 0.001	0.0	0.881	F
	Breast cancer	3	1.35(1.15, 1.59)	< 0.001	0.0	0.617	F
	TNBC	1	1.43(1.13, 1.80)	0.0003	–	–	–
	Other BC type	2	1.29(1.03, 1.60)	0.026	0.0	0.404	F
Follow-up	< 48 m	5	1.31(1.13, 1.52)	< 0.001	16.4	0.310	F
	> 48 m	2	1.41(1.19, 1.66)	< 0.001	0.0	0.822	F

*TNBC* triple-negative breast cancer, *RR* relative risk, *CI* confidence interval, *m* month, *F* fixed-effects, *P*_*A*_*P* value for association, *P*_*H*_*P* value for heterogeneity

### Meta-analysis for LVI

**Fig. 5:**
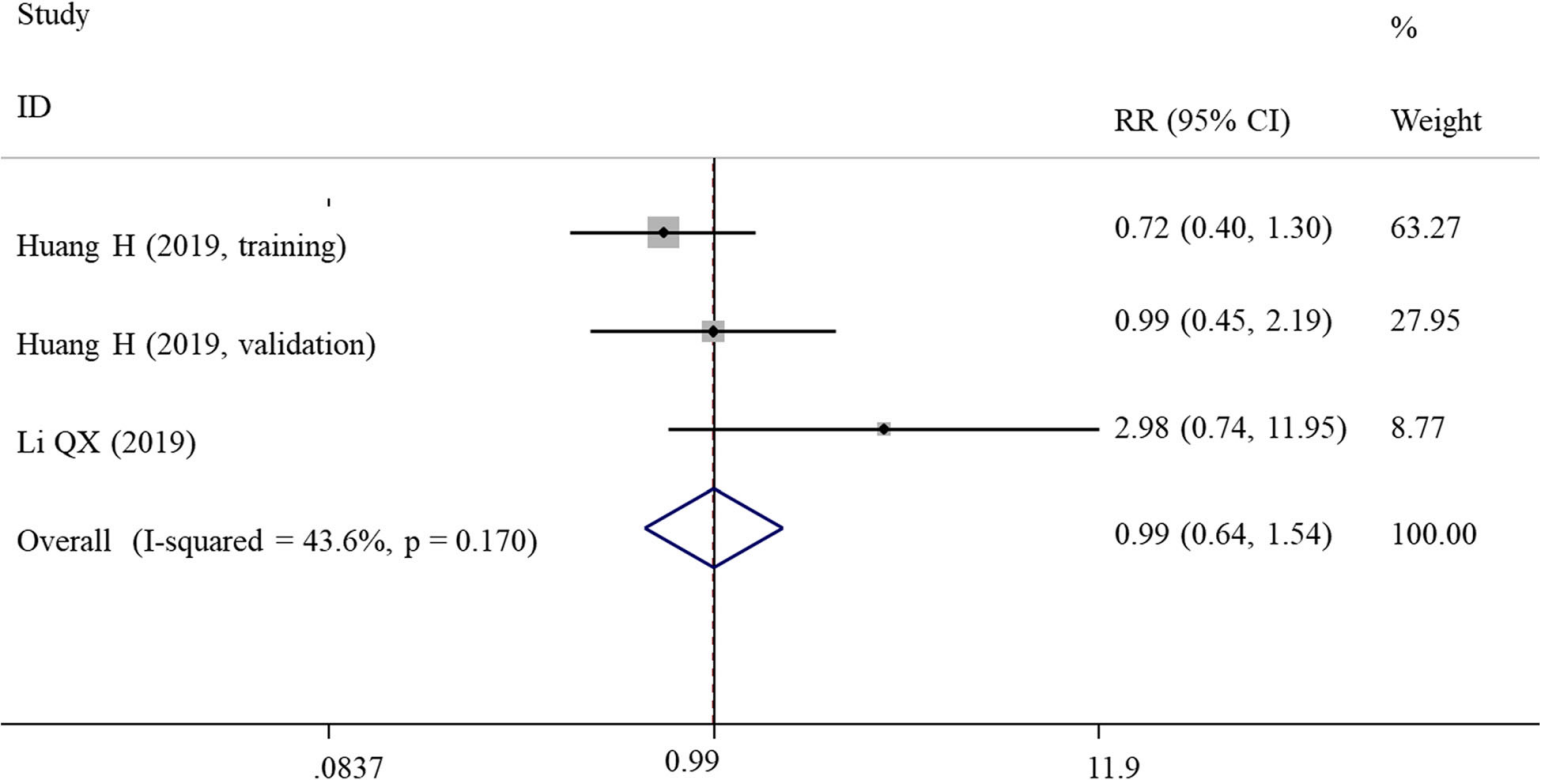
Forest plots showing the association between SII and lymphovascular invasion. SII, systemic immune-inflammation index; HR, hazard ratio; CI, confidence interval

Two studies with three datasets investigated the prognostic impact of SII on LVI. Meta-analysis using a fixed-effects model (*I*^2^ = 43.6%, *P* = 0.170) showed that SII index could not predict the LVI for patients with gynecological and breast cancers (RR = 0.99, 95% CI = 0.64–1.54; *P* = 0.972) (Fig. 5).

### Publication bias and sensitivity analyses

**Fig. 6:**
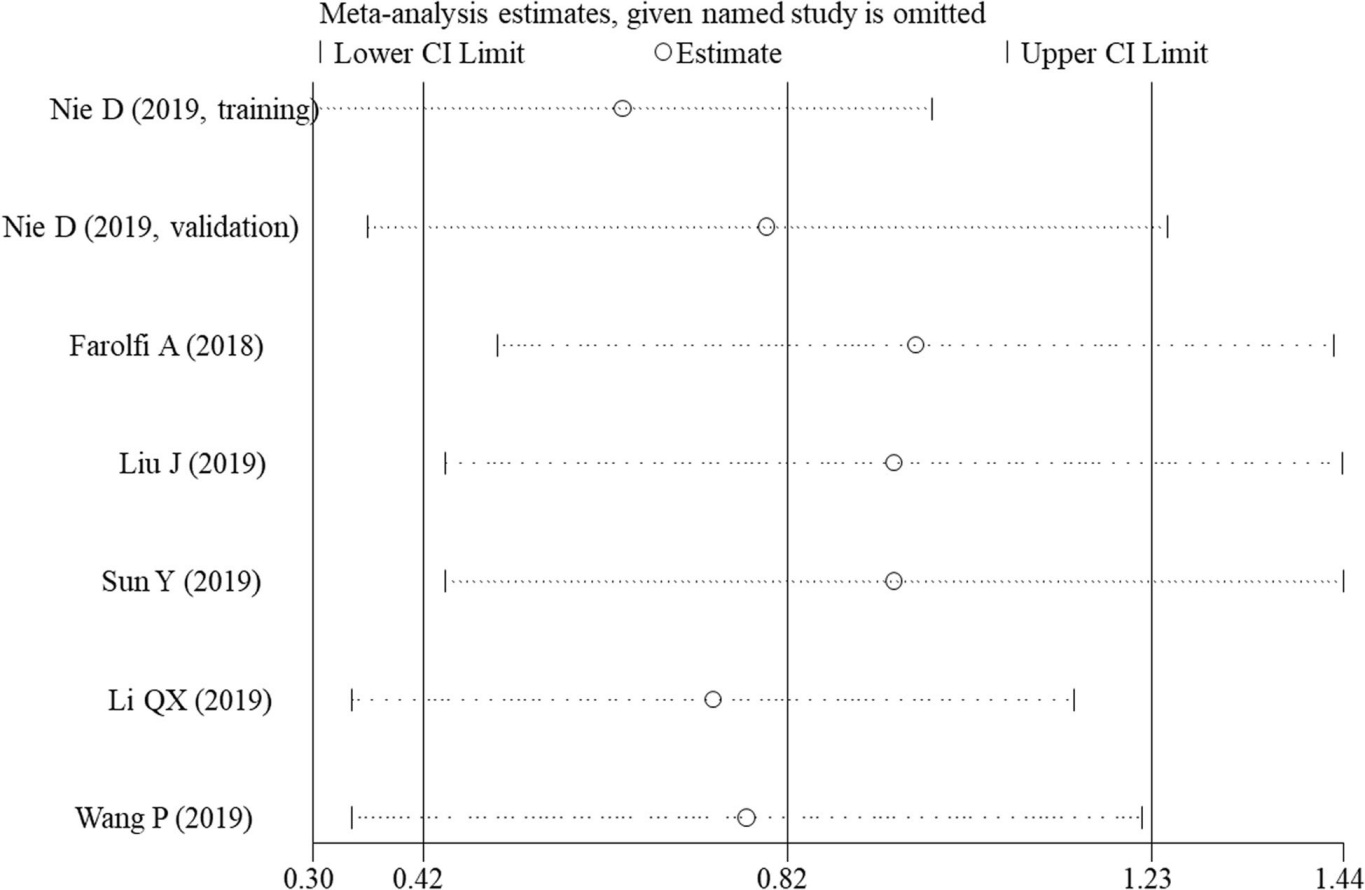
Sensitivity analysis for disease-free survival/progression-free survival. CI, confidence interval

Egger tests were carried out to assess the potential publication bias for studies with OS and DFS/PFS because obvious heterogeneities were seen among them as above described. The results showed that there was no evidence of publication bias for OS (*P* = 0.154). For DFS/PFS, publication bias seemed to be present (*P* = 0.007); however, the prognostic significance of SII remained unchanged (HR = 1.72, 95% CI = 1.112.67; *P* < 0.001) after trim-and-fill adjustment. The sensitivity analyses also demonstrated that the pooled results could not be affected after the removal of any one study (Fig. 6).

## Discussion

SII is a recently proposed new inflammatory index, which is calculated based on the count of neutrophils, platelets, and lymphocytes in the peripheral blood. Thus, SII may be effective to reflect the inflammatory status which is an important mechanism for the development of cancers and may be an underlying biomarker for prognosis prediction. This hypothesis had been demonstrated in meta-analyses on lung cancer [23, 24], esophageal cancer [25], gastrointestinal cancers [26], hepatocellular carcinoma [27], and several other cancer types [28, 29]. All these meta-analyses showed that increased SII predicted poor prognostic outcomes for patients with cancers. However, there was no study to confirm the prognostic roles of SII for gynecological and breast cancers that are two leading causes of death among women, which was the goal of our study. In line with the studies on other cancers [28, 29], we also found that elevated SII was associated with worse OS, DFS/PFS, and LNM of patients with gynecological and breast cancers compared with the low SII group. The conclusion on OS was applicable to all cancer types (cervical cancer, ovarian cancer, breast cancer), but the association with DFS/PFS and LNM was only significant for ovarian cancer and breast cancer, especially triple-negative breast cancer. These findings suggest that high SII may be a promising predictor for OS in patients with gynecological and breast cancers. For ovarian cancer and triple-negative breast cancer, SII may also serve as a useful prognostic indicator for their progression and survival.

Although the exact mechanisms remain poorly understood, the tumor-promoting functions of neutrophils and platelets, and the tumor-suppressing roles of lymphocytes may explain the prognostic values of high SII in cancers. For example, Coffelt et al. reported that tumor-induced neutrophils suppressed the activation of cytotoxic CD8+ T lymphocytes and then facilitated the establishment of metastases; while the absence of neutrophils profoundly reduced pulmonary and lymph node metastases of breast cancer cells [30]. Lee et al. demonstrated that ovarian tumor-derived neutrophils via forming neutrophil extracellular traps (NET) stimulated ovarian cancer cells colonized in the omentum to realize omental metastasis. Omental colonization and metastasis were found to be significantly decreased in mice with neutrophil-specific deficiency of peptidylarginine deiminase 4 (PAD4, an enzyme that is essential for NET formation) or undergoing the PAD4 inhibitor treatment (CI-amidine or GSK484) [31]. Yao et al. detected that the expression of tropomyosin 3 was significantly increased in platelets of patients with breast cancer compared with age-matched healthy controls. Overexpression of platelet tropomyosin 3 enhanced the migratory ability of breast cancer cells [32]. Hu et al. supported that platelet increased the growth of ovarian cancer in murine models due to high expression of transforming growth factor β1 (Tgfβ1); lack of platelet-specific Tgfβ1 in mice reduced tumor growth, neoangiogenesis, and platelet extravasation [33]. By co-incubation of platelets with breast or ovarian cancer cell lines, the study of Zuo et al. [34] and Guo et al. [35] directly proved that platelets exerted pro-metastatic functions via activation of epithelial-mesenchymal transition transformation. Thus, high levels of neutrophils/platelets and low levels of lymphocytes that led to an increased SII may ultimately contribute to the development and progression of gynecological (especially ovarian cancer) and breast cancers and related with poor prognosis of patients. Although several clinical trials [36] verified the prognostic roles of neutrophils, platelets, and lymphocytes for cervical cancer, rare in vitro and in vivo studies were performed to explore their functions in cervical cancer and further experiments are required.

There were some limitations that should be acknowledged. First, only 9 retrospective articles were included and the sample size for each cancer type was small. Also, there were none on endometrial cancer. Second, most of the studies were performed in China. There were studies to show that body mass index (BMI) varied by ethnicity and BMI was positively correlated with SII [37]. Hereby, ethnicity may affect SII and outcomes in patients with cancers. Third, the cut-off value varied in different articles. Fourth, most of the HRs and 95%CIs extracted from published articles were not adjusted by clinical related covariates. Therefore, the prognostic values of the SII in gynecological and breast cancers needed to be validated by using more trials with a prospective design, larger sample sizes, and patients from other countries.

## Conclusion

Our findings provide evidence that high SII may be a promising indicator for prediction of poor prognosis (OS, DFS/PFS, and LNM) in patients with gynecological and breast cancers, especially ovarian cancer and triple-negative breast cancer.

## Data Availability

All data generated or analyzed during this study are included in this published article.

## Abbreviations

NLR: Neutrophil-lymphocyte ratio
PLR: Platelet-lymphocyte ratio
SII: Systemic immune-inflammation index
OS: Overall survival
DFS: Disease-free survival
PFS: Progression-free survival
HR: Hazard ratio
CI: Confidence interval
AUC: Area under the curves
PRISMA: Preferred Reporting Items for Systematic Review and Meta-Analysis
NOS: Newcastle-Ottawa Scale
RR: Relative risk
LNM: Lymph node metastasis
LVI: Lymphovascular invasion
